# A novel closed reduction technique for treating femoral shaft fractures with intramedullary nails, haemostatic forceps and the lever principle

**DOI:** 10.1186/s12891-021-04055-5

**Published:** 2021-02-15

**Authors:** Wei Shui, Youyin Yang, Xinling Pi, Gang Luo, Bo Qiao, Weidong Ni, Shuquan Guo

**Affiliations:** 1 Department of Orthopaedic Surgery, The First Affiliated Hospital of Chongqing Medical University, 1 Youyi Rd, Chongqing, 400016 China; 2 Department of Orthopaedic Surgery, People’s Hospital of Chongqing Banan District, Chongqing, 401320 China; 3 Department of Nephrology, The First Affiliated Hospital of Chongqing Medical University, Chongqing, 400016 China

**Keywords:** Femoral shaft fracture, Closed fracture reduction, Internal fixation, Intramedullary nailing

## Abstract

**Background:**

Faster, easier, more economical and more effective versions of the minimally invasive reduction procedure for femoral shaft fractures need to be developed for use by orthopaedic surgeons. In this study, a fracture table was used to restore limb length, and long, curved haemostatic forceps and the lever principle were utilized to achieve minimally invasive reduction and intramedullary nail fixation of femoral shaft fractures.

**Methods:**

A retrospective analysis involving 20 patients with femoral shaft fractures reduced with a fracture table; long, curved haemostatic forceps; and the lever principle was conducted. The operative effect was evaluated on the basis of the operative time, reduction time, fluoroscopy time, and intraoperative blood loss.

**Results:**

All 20 cases were reduced in a closed fashion, and no conversions to open reduction were needed. The average operative time and fracture reduction time for all patients were 69.1 ± 13.5 min (range, 50–100 min) and 6.7 ± 1.9 min (range, 3–10 min), respectively. The fluoroscopy exposure time during the reduction process was 5–15 s, with an average time of 8.7 ± 2.7 s. The average intraoperative blood loss was 73.5 ± 22.5 mL (range, 50–150 mL). The patients exhibited excellent alignment in the injured limb after intramedullary nailing. Seventeen patients successfully completed a follow-up after fracture healing. The healing time ranged from 4 to 6 months.

**Conclusions:**

Displaced femoral shaft fractures in adults can be treated by a labour-saving lever technique involving fragments, 2 haemostatic forceps and soft tissue envelope-assisted closed reduction and intramedullary nail fixation. This technique is easy to perform; reduces blood loss, the fluoroscopy time and the surgical time for intraoperative reduction; and leads to excellent fracture healing.

## Background

Because it can preserve the soft tissue envelope, which is very important for fracture healing, intramedullary (IM) nailing is the first-line treatment and gold standard for femoral shaft fractures [1–3]. It is important that the fracture site is not directly exposed during the operation, as IM nailing was designed to be a minimally invasive treatment. Because the muscles around the femur are powerful and thick, both closed reduction and maintaining the reduction effectively during IM nail implantation are challenging for orthopaedic surgeons, and these procedures are experience dependent and require repeated attempts, resulting in a long duration of radiation exposure. The fracture table is widely used in surgery and is effective in restoring the length of the femur. However, the fracture table cannot achieve alignment independently [4]. Therefore, various devices have been introduced to facilitate closed reduction, including invasive devices for direct reduction, such as bone hooks, ball spikes, the finger reduction tool [4] and the Schanz pin (with the Joystick technique) [5, 6], as well as non-invasive methods for indirect reduction, such as F-tools [7], external support devices [8], rapid redactors [9, 10], and reduction frames [11]. These methods have their inherent advantages and disadvantages. However, given inter-individual differences among trauma patients, a large number of fracture patterns, regional differences among the medical facilities and technical skill level differences among surgeons, the repeatability and operability of these techniques are not ideal. Therefore, at present, no standard set of instruments is used for the closed reduction of femoral shaft fractures. Surgeons are trying to pursue faster, easier, more economical and more effective means of minimally invasive reduction, which remains challenging work.

We developed a novel simple method used for the minimally invasive reduction of femoral shaft fractures. On the basis of previously used methods of traction on the fracture table and the level principle with the soft tissue envelope serving as the fulcrum and long, curved haemostatic forceps serving as the lever arm, fractures of the femoral shaft can be reduced and maintained with a percutaneous minimally invasive method and finally fixed with an IM nail. Technical points, surgical outcomes, complication and other detailed information are provided in the following report.

## Methods

**Fig. 1:**
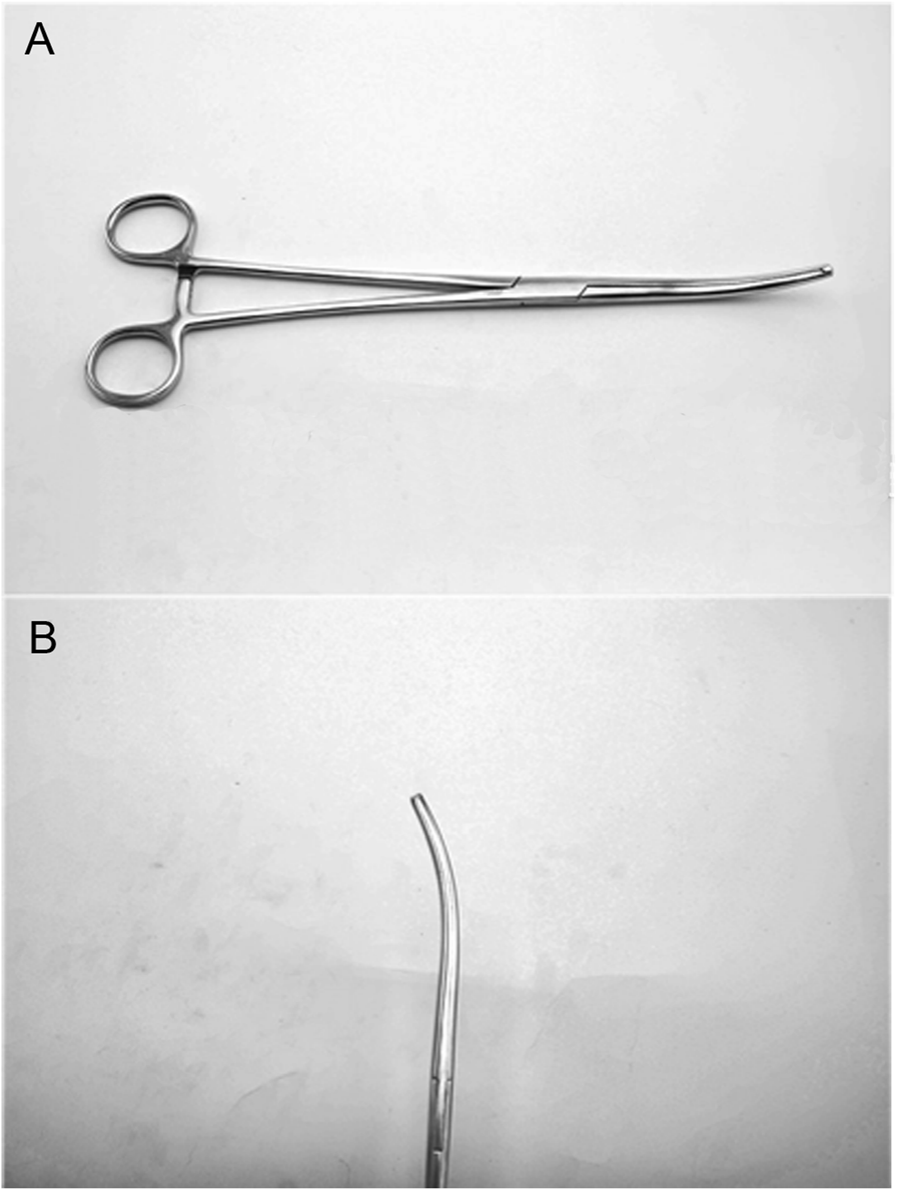
Photographs of the long, curved haemostatic forceps. **a** The length of the haemostatic forceps is 26 cm. **b** The side profile of the curved blunt tip

We used the largest size (26 cm) of the standard model of haemostatic forceps (J31346, JZ, Shanghai, China) in our operating room because these forceps have a curved blunt tip and are made of stainless steel (Fig. 1).

### Patients

A total of 20 patients were included in the present retrospective study and were treated surgically with this technique from March 2016 to January 2018. The inclusion criteria for the patients were as follows: 1. imaging findings confirming the presence of a displaced unilateral femoral shaft fracture without other fractures in the lower extremities and 2. an age > 18 years old. The exclusion criteria were open fractures, diabetes, prolonged steroid treatment, old fractures, and pathological fractures. All procedures were performed by the same group of surgeons. The 20 patients had an average age of 38 (range, 18–65) years and included 13 males and 7 females. According to the AO/OTA classification system, there were nine 32A cases, eight 32B cases and three 32C cases. Regarding laterality, 9 patients had fractures on the left side, and 11 patients had fractures on the right side. The interval from admission to the day of surgery was approximately 3–9 days with an average interval of 5.1 days. The study was approved by the ethics committee of the local hospital. Written informed consent was obtained from the patients for publication of identifying information in this research and any accompanying images.

### Operative technique with case demonstration (Figs. 2 and 3)

A 36-year-old male sustained a femoral shaft fracture (AO/OTA 32A3) after a traffic accident.

**Fig. 2:**
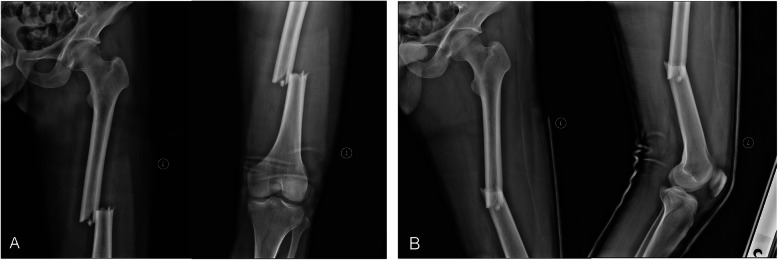
View of a 36-year-old male with a 32A3 fracture. **a** Preoperative anterior-posterior X-rays. **b** Preoperative lateral X-rays

**Fig. 3:**
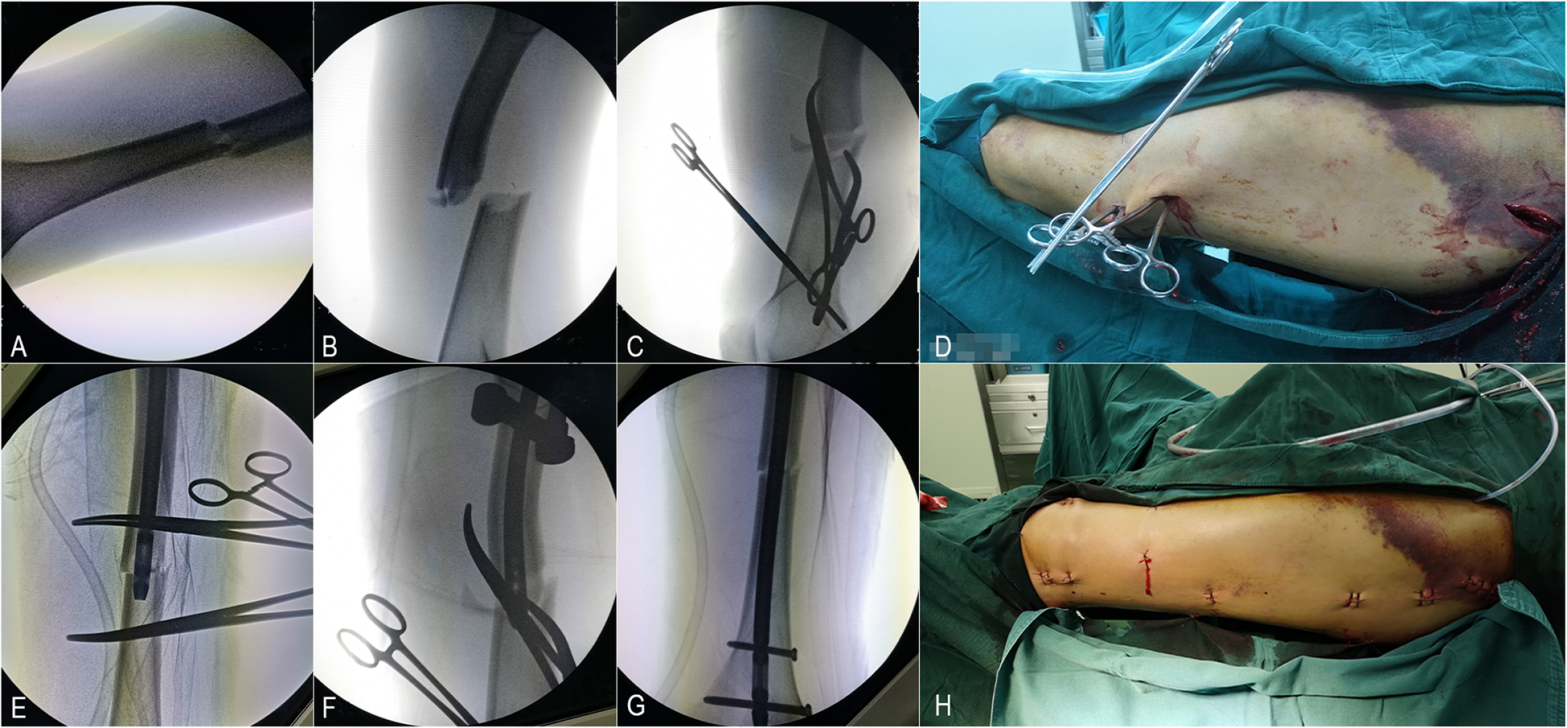
The view of the surgical procedure for this patient are shown in Fig. 2. **a** The “plane a” view showed that the shortening of the limb and lateral displacement of the fragment were nearly resolved after adducted traction on the fracture table. **b** The “plane b” view shows that the sagittal geometry was unstable and that the proximal fragment was flexed while the distal fragment sagged posteriorly. **c** The anterior-posterior displacement was reduced with two long, curved haemostatic forceps utilizing the lever principle. **d** The intraoperative photograph and **e** anterior-posterior and **f** lateral X-rays showed that the reduction was maintained by the 2 interlocking haemostatic forceps during the insertion of the IM nail. **g** After IM nailing. **h** Minimally invasive incision

After being anaesthetized, the patient was placed supine on the fracture table with the affected limb extended. The surgical assistant was instructed to adduct the affected limb and gradually increase the traction force until the degree of shortening was almost restored under the guidance of C-arm fluoroscopy (Fig. 3a).

**Fig. 4:**
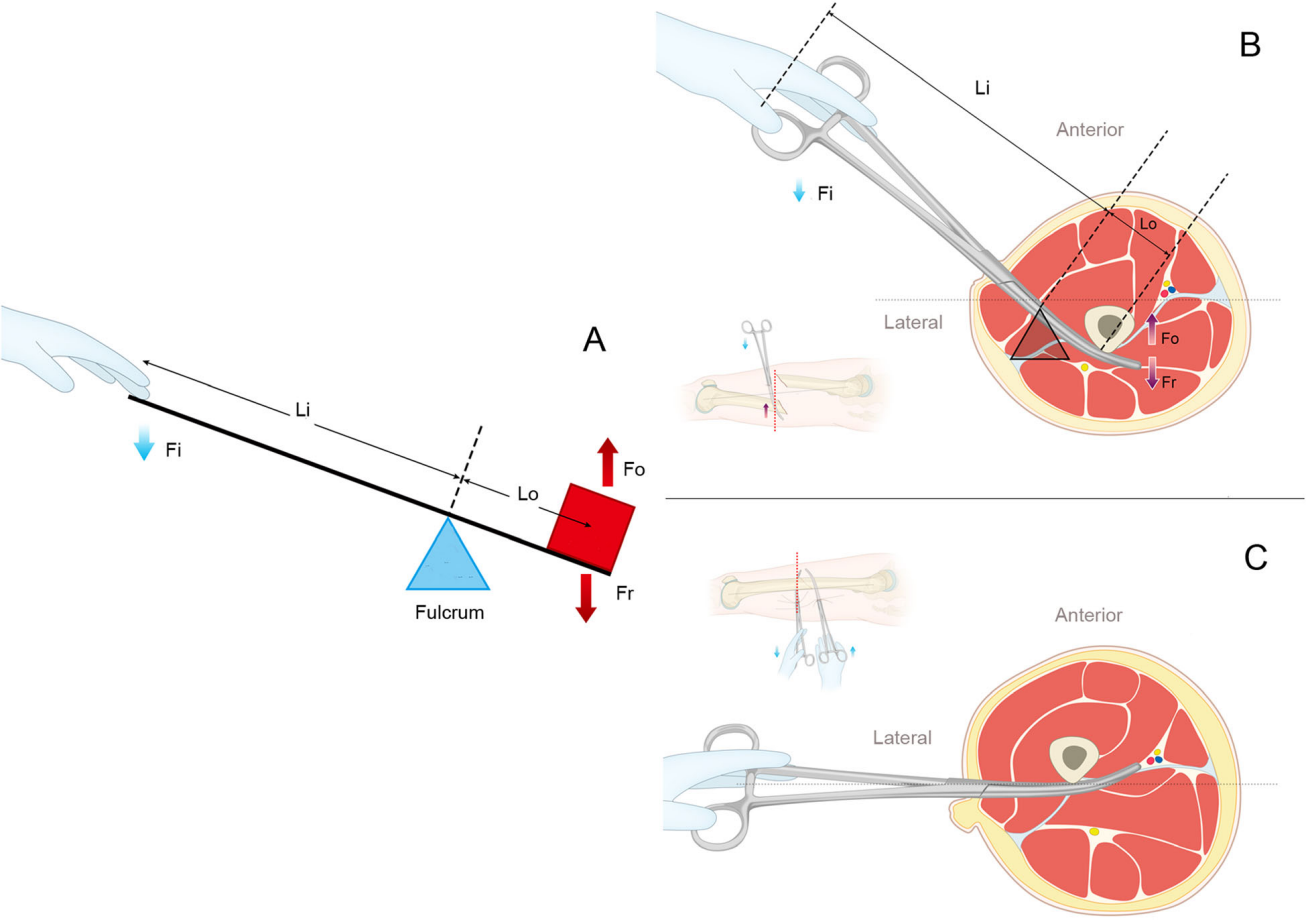
A schematic diagram of reduction with the lever principle. **a** The lever structure. **b** Cross-section of the distal fragment before reduction. **c** Cross-section of the distal fragment after reduction. Fi: in-force, Fo: out-force, Fr: resistance, Li: in-lever arm, Lo: out-lever arm

**Fig. 5:**
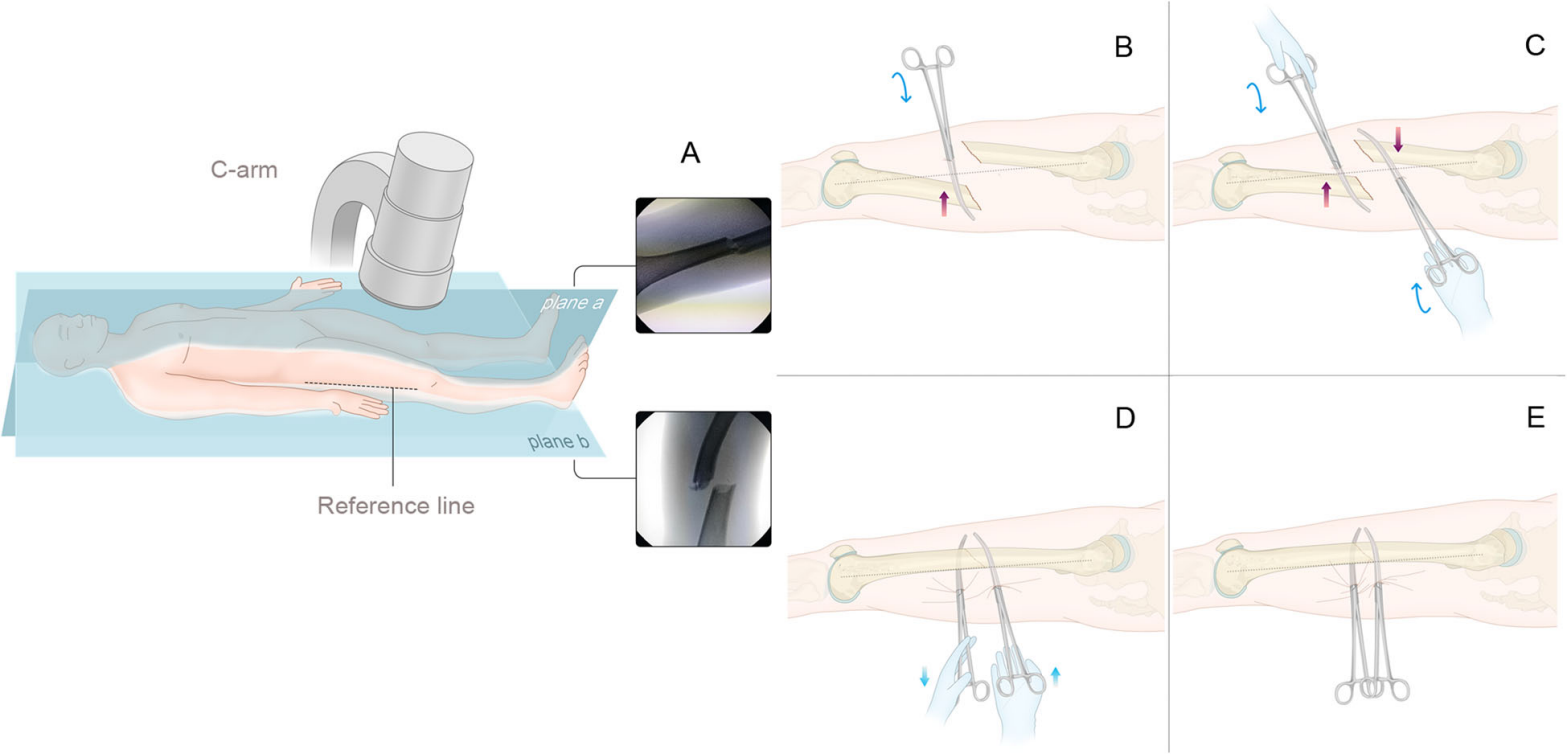
The reduction procedure with 2 haemostatic forceps utilizing the lever principle. **a** The C-arm fluoroscopic projection plane (plane a) shows that the proximal and distal fragments are almost aligned, whereas plane b, which is perpendicular to plane a, shows the anterior-posterior displacement. The reference line is the line intersecting plane b, travelling along the anatomical axis of the femur, and passing through the skin of the lateral thigh. Here, **b** and **c** show the position of the stab incision at the proximal fragment level and distal fragment level. **d** The proximal haemostatic forceps are used to lower the fragment and the distal forceps are used to elevate it to achieve reduction. **e** The 2 haemostatic forceps can be interlocked to maintain the reduction

The C-arm was used to confirm a fluoroscopic projection plane (plane a) in which the X-ray view shows that the fragments at the proximal and distal ends are nearly aligned (Fig. 3a) and a second plane (plane b) that is perpendicular to plane a and follows the anatomical axis of the femur (Fig. 3b). The line intersecting plane b and the skin of the lateral thigh is the reference line. A 0.5-cm incision was made at the distal fracture level and 1–2 cm anterior to the reference line. The first set of long, curved haemostatic forceps penetrated the muscle medially and posteriorly, touched the distal fragment, slid along the surface of the bone to the posterior side, and elevated the distal fragment, which was displaced posteriorly by lowering the haemostatic forceps. Another 0.5-cm incision was made at the proximal fracture level and 1–2 cm posterior to the reference line. A second set of long, curved haemostatic forceps penetrated the muscle medially and anteriorly, reached the proximal fragment, and then slid along the surface of the bone to the anterior side, lowering the proximal fragment, which was displaced anteriorly by elevating the haemostatic forceps (Figs. 3, 4 and 5). These 2 haemostatic forceps were interlocked with each other to maintain the reduction and were clamped by Kocher forceps to prevent them from sliding and needing to be held by the hand (Fig. 3d). The level of reduction was assessed by X-ray fluoroscopy. The cable method [12] and the linear relationship between the morphological alteration of the lesser trochanter and femoral rotation [13] were used for intraoperative evaluation of alignment. If satisfactory, the insertion and fixation procedures for the IM nail were performed using the standard method. The surgical assistant maintained the position of these 2 haemostatic forceps manually to prevent the loss of reduction resulting from the fracture site moving during the reaming and insertion processes for the IM nail. The operative time, reduction time, fluoroscopy time, and intraoperative blood loss were recorded.

### Postoperative management

On the second day after the operation, the patients were instructed to perform CPM-assisted passive function exercises of the affected limb. The patients who were capable of performing out-of-bed activities were encouraged to gradually start weight-bearing exercises with an assistive device 1 week after the operation.

Alignments in the frontal and sagittal planes were assessed on anteroposterior and lateral plain radiographs obtained immediately after surgery and at latest follow-up visits. Callus formation on 3/4 cortices and fading of fracture lines on radiographs were considered signs of fracture union [14].

## Results

### Surgical outcomes

All 20 cases of displaced femoral shaft fractures were reduced in a closed fashion, and no conversions to open reduction were needed. After IM nailing, the patients exhibited excellent alignment in the injured limb, and neither residual malreduction nor angular malalignment was detected in the fluoroscopic images. Iatrogenic neurovascular injury did not occur. The average operative time and fracture reduction time for all patients were 69.1 ± 13.5 min (range, 50–100 min) and 6.7 ± 1.9 min (range, 3–10 min), respectively. The fluoroscopy exposure time during the reduction process was 5–15 s with an average time of 8.7 ± 2.7 s. The average intraoperative blood loss was 73.5 ± 22.5 mL (range, 50–150 mL).

### Follow-up findings

**Fig. 6:**
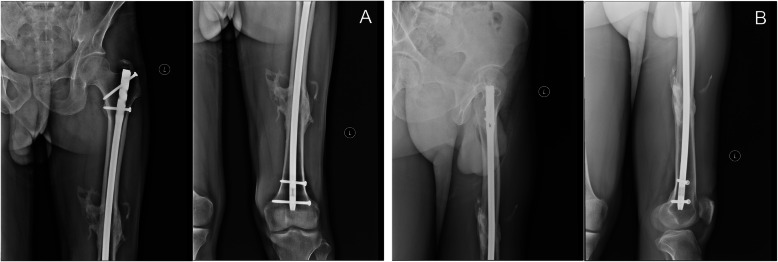
The view at 15 months postoperatively. **a** Anterior-posterior X-rays. **b** Lateral X-rays. Heterotopic ossification was observed

Of the 20 patients, 17 patients successfully completed a follow-up after fracture healing. The average follow-up time was 17.5 months (range, 15–20 months). All the patients who were followed up exhibited postoperative fracture healing; the healing time ranged from 4 to 6 months (Fig. 6). Deep venous thrombosis, breakage of internal fixation, malunion and infection were not observed.

## Discussion

Fractures of the femoral shaft are common high-energy injuries of the lower extremities [15]. Given the thick tissue envelope surrounding the femur, iatrogenic muscle injury and adhesion can be caused by conventional open reduction and internal fixation with the plate system [16]. The IM nail system makes it possible to achieve minimally invasive internal fixation and has become the gold standard in the management of fractures of the femoral shaft because it is associated with a high rate of union and a low complication rate [5, 17]. However, if the minimally invasive procedure cannot be achieved during the reduction process, minimally invasive internal fixation with an IM nail is not useful. The challenges associated with minimally invasive reduction result from the presence of numerous and powerful muscles attached to the femur, including the hip abductors and iliopsoas that are attached proximally, the adductors that are attached medially, and the gastrocnemius that is attached distally [18]. These muscles generate different displacement patterns of femoral shaft fractures. Incorrect fracture reduction can cause failure or breakage of the femoral nail [19]. Neutralizing the deforming forces of these muscles percutaneously and finally achieving minimally invasive reduction and fixation of femoral shaft fractures are the goals of surgeons.

Surgeons should not rely heavily on assistants; rather, they should be prepared to use instrumentation and positioning aids to facilitate reduction [5]. Fracture tables can be quite helpful in restoring the length of the lower limb by longitudinal traction but cannot restore the alignment directly; studies have shown that IM nailing of the femoral shaft performed without the use of a fracture table is significantly faster than that performed with a fracture table [4, 17]. Therefore, invasive tools that may facilitate reduction intraoperatively have been adopted, including the finger reduction tool [5] and Schanz pin [5, 6]. In our experience, the finger reduction tool can be used to control the proximal fragment but is challenging to aim at the entry site of the distal fragment; hence, fast and accurate reduction cannot typically be achieved. Moreover, the more distal the fracture site, the more difficult the finger reduction tool is to manipulate. When used as a “joystick”, the Schanz pin is theoretically associated with a risk of iatrogenic neurovascular injury and fibrosis or quadriceps contractures in the thigh [6]. In addition, with the aforesaid technique, the powerful muscles of the thigh must be overcome manually, which can result in an obviously laborious reduction process and difficulty maintaining the reduction. Furthermore, people involved in the operation, especially medical staff members, will be exposed to radiation during the entire process of reduction.

Various closed reduction devices have been developed for the treatment of femoral shaft fractures. Shezar et al. [8] established an external support device for the closed reduction of femoral shaft fractures but was unable to control the fragments in the coronal plane. Gao et al. [11] reported the application of a fracture-sustaining reduction frame for the closed reduction of femoral shaft fractures, but muscle swelling was observed due to compression of the frame. The “double reverse traction repositor” was developed by Zhang et al. [10] and is a type of rapid closed redactor that can function as the fracture table and has many advantages. However, the alignment of the fracture should be restored by other techniques. Additionally, the structure of the device was complex, and the assembly was time consuming [9, 10]. Zhu et al. [20] developed a teleoperated robot-assisted surgical system for the minimally invasive treatment of displaced femoral shaft fractures, but it is still an experimental model, which is predictably expensive and not ready for practical use [21]. Therefore, given these technical difficulties, many surgeons continue to consider open reduction and internal fixation [22] but do not consider the advantages of IM nailing.

The lever principle is an ancient theory of mechanics that was proposed by Chinese scientist Mozi and Greek scientist Archimedes as early as third century BC [23]. Levers can be used to exert a large force over a short distance at one end and only a small force over a longer distance at the other end. We used the lever principle in the present study. After the shortened limb was lengthened by the fracture table, a labour-saving lever structure was assembled using the long, curved haemostatic forceps serving as the lever arm and the soft tissue serving as the fulcrum (Fig. 4). All the manipulations were performed percutaneously. Only a small force exerted by a surgeon can counteract the retracting force of the thigh muscles to easily achieve closed reduction of femoral shaft fractures. The technical points are as follows: (1) Sufficient traction and restoration of the limb length need to be achieved first, as they are prerequisites for the subsequent steps. In our study, these steps were achieved by the fracture Table. (2) Moderate adduction of the affected lower limb on the fracture table can partially neutralize the deforming forces of the adductors, which allows femoral shaft fractures to be reduced laterally and the antegrade IM nail to be inserted. (3) The tension resulting from the traction makes the soft tissue envelope sufficiently rigid to serve as a lever fulcrum, and this process cannot be achieved when the muscles are relaxed. Moreover, the closed soft tissue envelope can compact the fragments and restrict their movement with the tension of the muscles, which is conducive for reduction. (4) Because femoral shaft fracture cases differ across individuals, the displacement patterns of the fracture site vary after traction on the fracture table. Nevertheless, regardless of the displacement pattern, there is a C-arm fluoroscopic projection plane (plane a) in which the X-ray view shows that the fragments at the proximal and distal ends are approximately aligned and a second plane (plane b) that is perpendicular to plane a and follows the anatomical axis of the femur. The line intersecting plane b and the skin of the lateral thigh is the reference line. The 2 haemostatic forceps were inserted on either side of the reference line (Fig. 5a). In our study, most cases were approximately reduced laterally after longitudinal traction. For these patients, plane a was the approximate sagittal plane, plane b was the approximate coronal plane, and the reference line was the approximate midline of the lateral thigh (Fig. 5a). (5) We chose to use 26-cm curved haemostatic forceps because haemostatic forceps of this size are sufficiently thick and rigid to serve as excellent lever arms. Moreover, the forceps are of a sufficient length. Shorter forceps would not be able to serve as a lever arm and thus could not reduce the labour required, and surgeons would not be able to control the fragment as well if the forceps are too long. Furthermore, the curved blunt tip of the forceps restricts the fragment from slipping during the reduction process and allows the surgeon to make small adjustments to determine the best position for reduction.

**Fig. 7:**
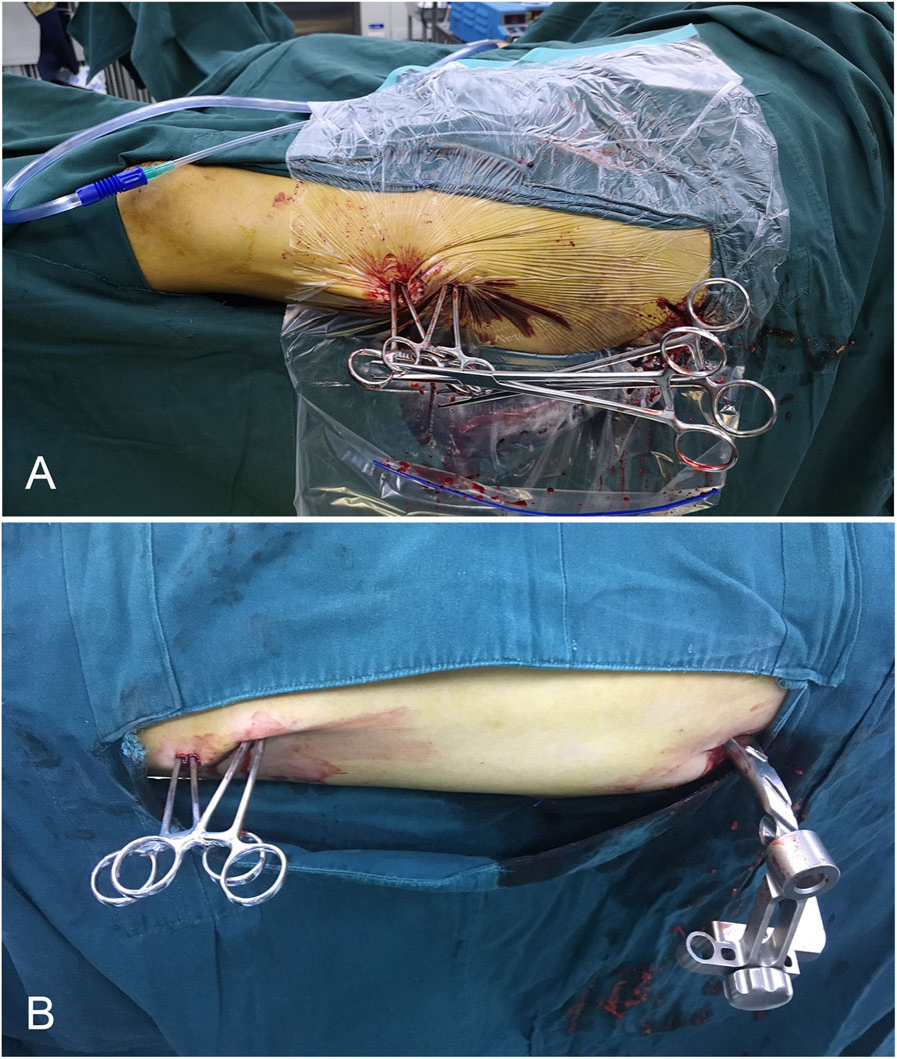
The two haemostatic forceps can be interlocked with each other, forming an automatic stable structure that can maintain the reduction without a manual operation. **a** The Kocher forceps were used to prevent sliding. **b** Under ideal conditions, the haemostatic forceps can remain stable without Kocher forceps

In terms of the 20 patients with femoral shaft fractures who underwent reduction with long, curved haemostatic forceps, reduction was successful, and the results were satisfactory. Long, curved haemostatic forceps are readily available and inexpensive. The labour-saving lever structure constituted by fragments, haemostatic forceps and a soft tissue envelope can facilitate reduction. Because the resistance from the proximal and distal fragments are in opposing directions, when reduction is completed by lowering the proximal fragment and elevating the distal fragment, the 2 haemostatic forceps can interlock with each other. This feature facilitates neutralization of the resilience force and reduction of the risk of reduction loss, leading to an automatically stable construct that can maintain the reduction effect without manual operations (Figs. 5e and 7) and allowing the surgeon to stand at a distance during the fluoroscopic monitoring of the fracture site. In contrast, when the Schanz pin is used as a “joystick”, the bone structure will not be injured, and iatrogenic secondary fractures may be avoidable. Ideally, the haemostatic forceps penetrate the muscle fibres rather than sever them through just two 0.5-cm incisions. The degree of muscle injury is slight, and complete minimally invasive closed reduction can be achieved. Although perfect alignment cannot be achieved in most cases, it is sufficient to allow the insertion of an IM nail. This is a simple technique with a high operative speed, a short operative time, a short radiation exposure time, and a short learning curve. In addition, this technique can be mastered by most surgeons in a short time. In the present study, the average reduction time, operative time, fluoroscopy time and blood loss were 6.7 ± 1.9 min, 69.1 ± 13.5 min, 8.7 ± 2.7 s and 73.5 ± 22.5 mL, respectively, which were lower than the values reported in studies on other minimally invasive techniques, such as the “double reverse traction repositor” [9] and fracture-sustaining reduction frame techniques [11].

In our experience, the best indications of this technique are fracture patterns with mainly anterior-posterior and lateral displacement and a small degree of rotation. Given the external rotation, abduction, and flexion displacement of the proximal fragment in subtrochanteric fractures, it is difficult to achieve reduction using this technique alone, and the use of a second reduction tool, such as a ball spike, the Schanz pin and a periosteal elevator, may be necessary. We did not apply this technique to retrograde nail because the tension resulting from the traction play a very important role in our reduction process, and the reduction is always achieved by flexing the knee to reduce the muscle force in the retrograde nail fixation process [24]. Heterotopic ossification was observed during the follow-up period (Fig. 6), which may be related to the local haematoma at the fracture site that could not be debrided during the closed reduction and fixation procedures. Nonetheless, limb function is not affected by heterotopic ossification distant from the joint.

The small sample size and the absence of a control group for the comparison of outcomes are the limitations of this study. However, there is no recognized gold standard for the minimally invasive reduction of femoral shaft fractures, so it is difficult to design an appropriate control group.

In conclusion, displaced femoral shaft fractures in adults can be treated by lever principle-assisted closed reduction and IM nail fixation. The labour-saving lever structure constituted by fragments, 2 haemostatic forceps and a soft tissue envelope can both reduce displaced femoral shaft fractures and maintain reduction in an anatomical position, which enables IM nailing fixation to be successfully achieved. This technique is easy to perform; reduces blood loss, the fluoroscopy time and the surgical time for intraoperative reduction; and leads to excellent fracture healing.

## Data Availability

The datasets concerning this study are available from the corresponding author on reasonable request.

## Abbreviations

AO/OTA: AO Foundation/Orthopaedic Trauma Association
IM: Intramedullary
CPM: Continuous passive motion
